# Cephalopod-omics: Emerging Fields and Technologies in Cephalopod Biology

**DOI:** 10.1093/icb/icad087

**Published:** 2023-06-27

**Authors:** Tom Baden, John Briseño, Gabrielle Coffing, Sophie Cohen-Bodénès, Amy Courtney, Dominick Dickerson, Gül Dölen, Graziano Fiorito, Camino Gestal, Taryn Gustafson, Elizabeth Heath-Heckman, Qiaz Hua, Pamela Imperadore, Ryosuke Kimbara, Mirela Król, Zdeněk Lajbner, Nicolás Lichilín, Filippo Macchi, Matthew J McCoy, Michele K Nishiguchi, Spencer V Nyholm, Eve Otjacques, Pedro Antonio Pérez-Ferrer, Giovanna Ponte, Judit R Pungor, Thea F Rogers, Joshua J C Rosenthal, Lisa Rouressol, Noelle Rubas, Gustavo Sanchez, Catarina Pereira Santos, Darrin T Schultz, Eve Seuntjens, Jeremea O Songco-Casey, Ian Erik Stewart, Ruth Styfhals, Surangkana Tuanapaya, Nidhi Vijayan, Anton Weissenbacher, Lucia Zifcakova, Grace Schulz, Willem Weertman, Oleg Simakov, Caroline B Albertin

**Affiliations:** School of Life Sciences, University of Sussex, Brighton BN1 9QG, UK; Molecular and Cell Biology Department, University of Connecticut, Storrs, CT 06269, USA; Biology Department: Institute of Ecology and Evolution, University of Oregon, Eugene, OR 97403-5289, USA; Laboratoire des Systèmes Perceptifs, Département d'Etudes Cognitives, Ecole Normale Supérieure, PSL University, CNRS, 75005 Paris, France; MRC Laboratory of Molecular Biology, Francis Crick Avenue, Cambridge CB2 0QH, UK; Friday Harbor Laboratory, University of Washington, Seattle, WA 98250, USA; Department of Neuroscience, Johns Hopkins University, Baltimore, MD 21218, USA; Department of Biology and Evolution of Marine Organisms, Stazione Zoologica Anton Dohrn, 80121 Napoli, Italy; Laboratory of Marine Molecular Pathobiology, Institute of Marine Research (IIM), Spanish National Research Council (CSIC), Vigo 36208, Spain; University of Central Florida, Orlando, FL 32826, USA; Departments of Integrative Biology and Microbiology and Molecular Genetics, Michigan State University, East Lansing, MI 48824, USA; Department of Ecology and Evolution, University of Adelaide, Adelaide, South Australia 5000, Australia; Department of Biology and Evolution of Marine Organisms, Stazione Zoologica Anton Dohrn, 80121 Napoli, Italy; Misaki Marine Biological Station, School of Science, The University of Tokyo, Miura, Kanagawa 238-0225, Japan; Adam Mickiewicz University in Poznań, Poznań 61-712, Poland; Physics and Biology Unit, Okinawa Institute of Science and Technology Graduate University, 1919-1 Tancha, Onna, Kunigami District, Okinawa 904-0495, Japan; Department of Neurosciences and Developmental Biology, University of Vienna, Vienna 1010, Austria; Program in Biology, New York University Abu Dhabi, P.O. Box 129188 Abu Dhabi, United Arab Emirates; Department of Pathology, Stanford University, Stanford, CA 94305, USA; Department of Molecular and Cell Biology, School of Natural Sciences, University of California, Merced, 5200 N. Lake Blvd., Merced, CA 95343, USA; Molecular and Cell Biology Department, University of Connecticut, Storrs, CT 06269, USA; MARE—Marine and Environmental Sciences Centre & ARNET—Aquatic Research Network, Laboratório Marítimo da Guia, Faculdade de Ciências, Universidade de Lisboa, Av. Nossa Senhora do Cabo, 939, 2750-374 Cascais, Portugal; Division of Biosphere Sciences and Engineering, Carnegie Institution for Science, 1200 E. California Blvd, Pasadena, CA 91125, USA; Department of Molecular and Cell Biology, School of Natural Sciences, University of California, Merced, 5200 N. Lake Blvd., Merced, CA 95343, USA; Department of Biology and Evolution of Marine Organisms, Stazione Zoologica Anton Dohrn, 80121 Napoli, Italy; Biology Department: Institute of Ecology and Evolution, University of Oregon, Eugene, OR 97403-5289, USA; Department of Neurosciences and Developmental Biology, University of Vienna, Vienna 1010, Austria; Marine Biological Laboratory, The Eugene Bell Center for Regenerative Biology and Tissue Engineering, Woods Hole, MA 02543-1015, USA; Department of Neurosciences and Developmental Biology, University of Vienna, Vienna 1010, Austria; Department of Molecular Biosciences and Bioengineering, University of Hawaii Manoa, Honolulu, HI 96822, USA; Molecular Genetics Unit, Okinawa Institute of Science and Technology Graduate University, Onna, Okinawa 904-0495, Japan; MARE—Marine and Environmental Sciences Centre & ARNET—Aquatic Research Network, Laboratório Marítimo da Guia, Faculdade de Ciências, Universidade de Lisboa, Av. Nossa Senhora do Cabo, 939, 2750-374 Cascais, Portugal; Department of Neurosciences and Developmental Biology, University of Vienna, Vienna 1010, Austria; Laboratory of Developmental Neurobiology, Department of Biology, KU Leuven, Leuven 3000, Belgium; Biology Department: Institute of Ecology and Evolution, University of Oregon, Eugene, OR 97403-5289, USA; Neural Circuits and Behaviour Lab, Max‐Delbrück‐Center for Molecular Medicine in the Helmholtz Association (MDC), Berlin 13125, Germany; Laboratory of Developmental Neurobiology, Department of Biology, KU Leuven, Leuven 3000, Belgium; Laboratory of genetics and applied breeding of molluscs, Fisheries College, Ocean University of China, Qingdao 266100, China; Molecular and Cell Biology Department, University of Connecticut, Storrs, CT 06269, USA; Vienna Zoo, Maxingstraße 13b, 1130 Vienna, Austria; Physics and Biology Unit, Okinawa Institute of Science and Technology Graduate University, 1919-1 Tancha, Onna, Kunigami District, Okinawa 904-0495, Japan; University of Chicago, Chicago, IL 60637, USA; Friday Harbor Laboratory, University of Washington, Seattle, WA 98250, USA; Department of Neurosciences and Developmental Biology, University of Vienna, Vienna 1010, Austria; Marine Biological Laboratory, The Eugene Bell Center for Regenerative Biology and Tissue Engineering, Woods Hole, MA 02543-1015, USA

## Abstract

Few animal groups can claim the level of wonder that cephalopods instill in the minds of researchers and the general public. Much of cephalopod biology, however, remains unexplored: the largest invertebrate brain, difficult husbandry conditions, and complex (meta-)genomes, among many other things, have hindered progress in addressing key questions. However, recent technological advancements in sequencing, imaging, and genetic manipulation have opened new avenues for exploring the biology of these extraordinary animals. The cephalopod molecular biology community is thus experiencing a large influx of researchers, emerging from different fields, accelerating the pace of research in this clade. In the first post-pandemic event at the Cephalopod International Advisory Council (CIAC) conference in April 2022, over 40 participants from all over the world met and discussed key challenges and perspectives for current cephalopod molecular biology and evolution. Our particular focus was on the fields of comparative and regulatory genomics, gene manipulation, single-cell transcriptomics, metagenomics, and microbial interactions. This article is a result of this joint effort, summarizing the latest insights from these emerging fields, their bottlenecks, and potential solutions. The article highlights the interdisciplinary nature of the cephalopod-omics community and provides an emphasis on continuous consolidation of efforts and collaboration in this rapidly evolving field.

## Introduction

**Fig. 1:**
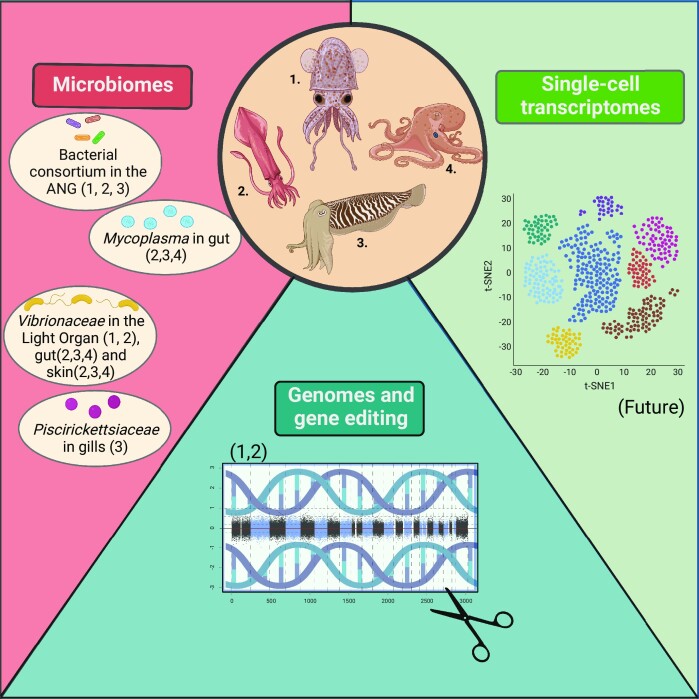
Research questions addressable with emerging technologies for cephalopods. Numbers in brackets refer to the numbers assigned to the different types of cephalopods. (1) Bobtail squid, (2) Loliginid squid, (3) Cuttlefish, and (4) Octopus (Created with BioRender.com).

Cephalopods (squid, octopus, cuttlefish, and nautilus) are a molluscan class that is subdivided into coleoid cephalopods (squid, octopus, and cuttlefish) and nautiloids (e.g., *Nautilus*). Ten years have passed since the publication of the first cephalopod genomics white paper (Albertin et al. 2012). While it still took a few years until the first cephalopod genome was released (Albertin et al. 2015), cheap sequencing costs, and improvement of assembly algorithms have since resulted in an accelerated pace of cephalopod genomics studies (Albertin and Simakov 2020). Furthermore, technological advances have allowed for the development of new fields in cephalopod molecular biology, from unraveling aspects of gene regulation (Schmidbaur et al. 2022) to single cell transcriptomics (Duruz et al. 2022 ; Gavriouchkina et al. 2022; Songco-Casey et al. 2022; Styfhals et al. 2022) to targeted gene manipulation (Crawford et al. 2020). This summary of the state-of-the-art and future direction in cephalopodomics is a result of a collaboration of more than 40 authors who met at a workshop at the Cephalopod International Advisory Council (CIAC) conference in Portugal in 2022, preceded by online meetings within the CephRes Virtual Event in 2020. Our goal was to identify major developments in the field and emerging bottlenecks, and outline major ongoing directions, as well as possible solutions to arising problems. We focused on four major topics that were discussed at the workshop, spanning comparative and regulatory genomics, transgenics, gene expression and single cell transcriptomics, and metagenomics, with the results and views of each of the working groups presented below (Fig. 1). These topics emerged from group discussions focused on outlining the current state of the field, as well as developing emerging and future approaches for use in studying cephalopod biology. Being a small subset of the whole community, and due to space limitations, we cannot cover all possible topics of cephalopod molecular research or go in too much depth even in the selected set of research areas. We thus apologize to colleagues whose work we could not represent sufficiently here, and for this we refer the reader to the more specialized review articles.

## Resources and emerging new standards for cephalopod genomics

### Sequencing strategy proposal

The diversity and quality of published cephalopod genome assemblies have increased considerably in the 7 years since the publication of the first cephalopod genome, that of *Octopus bimaculoides* (Albertin et al. 2015). The accelerated pace of chromosomal-scale assembly and chromosomal-level genomic comparisons allows for new topics to be explored. Major emerging topics include: chromosomal evolution (Albertin et al. 2022), first insights into regulatory regions (Schmidbaur et al. 2022), gene architecture and non-coding element evolution (McCoy and Fire 2020; Heath-Heckman and Nishiguchi 2021; Marino et al. 2022), phylogenetics (Tanner et al. 2017; Sanchez et al. 2021), among others. These studies have so far shown that coleoid genomes are typically large, often spanning ∼3–6 Gb across 30 chromosomes for octopuses and ∼46 for squids, while many molluscan genomes are less than 2 Gb (genomesizes.com). While the number of genes in cephalopods are similar to many other invertebrates, the non-coding genome appears to be significantly larger (Albertin et al. 2015; McCoy and Fire 2020), with at least half of a typical cephalopod genome composed of repetitive elements (Marino et al. 2022) and an increased number of miRNAs (Zolotarov et al. 2022). The large and highly repetitive nature of these genomes necessitates rigorous approaches in genome assembly and annotation, similar to what is required for many vertebrate genomes.

Many genome sequencing consortia have developed standardized approaches to genome sequencing and assembly (Rhie et al. 2021). The current “gold standard” of cephalopod genomics is long read (PacBio, Oxford Nanopore) sequencing of around 25–40× coverage using HiFi (High-Fidelity) or at least 50× CLR (Continuous Long Reads) (McKenna et al. 2021). The first major community goal is thus to produce chromosome-scale and phased genome assemblies of cephalopods, which provides haplotype information to help untangle relationships between DNA sequence and phenotype. Resolving the genomes to chromosome-scale and phasing the haplotypes additionally requires chromosomal conformational data (Hi-C), or preferably the more recent derivative DNAse Hi-C (Ma et al. 2015), from the same individual from which the long reads are derived. Recent algorithmic advancements integrate high-fidelity PacBio reads (HiFi), ultra-long nanopore reads, and phasing information from Hi-C libraries to generate telomere-to-telomere assemblies and should be considered for cephalopods (Cheng et al. 2021; Nurk et al. 2022). Alternatively, inbred individuals could be sequenced to decrease the assembly graph complexity introduced by the frequent heterozygous sites in cephalopod genomes (Albertin et al. 2015).

### Towards high-quality genome annotations

The second major goal is producing high-quality genome annotations. Standard annotation pipelines, such as MAKER (Cantarel et al. 2008), AUGUSTUS (Hoff and Stanke 2019), or BRAKER (Brůna et al. 2021), produce sufficient quality gene models for basic orthology and evolutionary studies. However, the use of standard Illumina RNA-seq reads can lead to several problems in genome annotations, including the lack of 3′ and 5′ UTRs, fragmented or fused gene models, and poor gene calling ability in repeat-rich regions. This can be particularly problematic for read mapping in single-cell RNA-seq data, which is commonly 3′ biased (see below). Contemporary genome annotation pipelines, such as MAKER with ProtHint (Brůna et al. 2020) incorporation, can facilitate the annotation process by using protein orthology information and full-length transcript sequences from PacBio Iso-Seq or Oxford Nanopore Technologies R2C2. However, we expect that the large cephalopod genomes will require further manual curation to correct inappropriately fused or fragmented gene models.

To facilitate gene annotation of the many emerging cephalopod systems, the community goal is to generate high-quality annotations for a particular subset of species. There are several considerations for selecting among current coleoid cephalopods, including available resources, individual community sizes, and long-term outlook for transgenics. Ideally, these efforts should be distributed across the two major clades of coleoids: the Decapodiformes (the clade including bobtails, squid, and cuttlefish), such as *Euprymna scolopes* and *Euprymna berryi, Sepioteuthis lessiona*, and the Octopodiformes (vampire squid, octopuses, and their kin), such as *Octopus vulgaris* and *O. bimaculoides* (Albertin et al. 2020). This will allow the community to produce a high-quality set of curated orthologs—which we propose to term CephUSCO (single copy orthologs)—for curation of follow-up models, similar to efforts completed in other clades, e.g., mollusks (Caurcel et al. 2021). Additionally, standardized gene naming is crucial for effective communication about genes (Bruford et al. 2020). Presently, we suggest that the cephalopod community follow the naming convention proposed for human genes (https://vertebrate.genenames.org/about/, the HUGO Gene Nomenclature Committee (HGNC), while efforts to characterize the function of cephalopod genes are ongoing.

These recent advancements have made sequencing and assembling cephalopod genomes to chromosome-scale, then annotating them, easier for the growing cephalopod research community (e.g., Destanović et al. 2023). The increased amount of community genomic resources will help with the continued development of nascent model systems for molecular research (see below) as well as the investigation of poorly studied or rare cephalopod species (McKenna et al. 2021; Vecchione et al. 2022).

## Towards cephalopod transgenics and regulatory genomics

The historical success and persisting popularity of the major invertebrate model organisms, *C. elegans* and *D. melanogaster*, are due, in part, to their genetic tractability. A genetically tractable organism is a research organism amenable to genetic modifications, i.e., the genetic makeup of the organism is altered to achieve a desired outcome. Genetic tractability is essential as it allows researchers to functionally test correlative genotype–phenotype observations and opens up the possibility of creating tools, like reporter lines, that will enable new areas of study. Well-established methods in other model organisms enable us to remove (knock-out) or insert (knock-in) specific genomic regions, or cause a downregulation (knock-down) or upregulation of expression (over-expression). Knocking out a gene of interest and observing phenotypic changes allows us to understand the role that gene plays. Knocking in fluorescent proteins under cell type specific promoters enables us to visualize expression patterns on a cellular or protein level. For neurobiological studies, cell-type-specific knock-in of neural activity reporters (e.g., fluorescent calcium indicators like GCaMP) allows us to monitor the activation of specific neurons even while the animal is behaving (Broussard et al. 2014). Progress applying these tools in other emerging model organisms, such as the cnidarian *Hydra vulgaris*, the acoel *Hofstenia miamia*, the hemichordate *Saccoglossus kowalevskii* (Minor et al. 2019), and crustaceans like *Parhyale hawaiiensis*, was made possible after sequencing breakthroughs and development of transgenic lines (Pavlopoulos et al. 2005; Badhiwala et al. 2021; Ricci et al. 2021). Developing a genetically tractable organism requires a species that can be easily cultured in the lab, has high-quality genomic and transcriptomic data, and has reliable transgene injection techniques. The following sections will discuss the progress the cephalopod community has made in these respects and what is still required to enable a fully genetic tractable cephalopod species.

### Goals and challenges for gene manipulation in cephalopods

While gene manipulation tools have been developed in a number of emerging model systems in many animal clades, these methods have only recently becoming available in cephalopods. Thus far, CRISPR-mediated knockouts have only been demonstrated for *Doryteuthis pealeii* (Crawford et al. 2020), and transgenics have yet to be reported in any cephalopod. However, efforts are underway to develop transgenics in all of the genome-enabled systems (see above), in particular for the Japanese bobtail *Euprymna berryi* (Ahuja et al. 2023), the Hawaiian bobtail squid *Euprymna scolopes*, as well as the stumpy cuttlefish *Sepia bandensis*, although other species may become readily available in the future as well (see below). In general, major technical obstacles to achieve transgenesis include introducing reagents into a cell, identifying robust and reproducible methods for integrating exogenous DNA, and driving transgene expression (transiently or integrated). These barriers have been overcome in several other emerging animal models (Pavlopoulos et al. 2005; Minor et al. 2019; Badhiwala et al. 2021; Ricci et al. 2021), and incorporating approaches and reagents that have been successful in these other species may accelerate the development of these technologies in cephalopods.

### Delivery timing and injection techniques are species-specific

A number of methods have been established in other model organisms to introduce reagents into a cell, including microinjection, electroporation, transfection, and viral vectors. Thus far, the only published method for introducing reagents into cephalopod cells is microinjection (Crawford et al. 2020). This method requires routine access to early cleavage-stage embryos and means of accessing the early blastomeres. Delivery techniques for CRISPR guides vary depending on the species. Naturally laid decapodiform embryos typically have a number of extraembryonic structures protecting the developing embryo, including a chorion, jelly layers, and an outer tunic. These structures differ between different groups, and each presents a barrier to accessing embryonic cells. For example, naturally laid *Doryteuthis* embryos have a tough chorion surrounded by jelly layers and an outer tunic that joins embryos into “fingers.” However, *in vitro*-fertilized *Doryteuthis* embryos lack the outer jelly layers and tunic (Crawford 2002; Crawford et al. 2020), allowing timed experiments. However, they do have a thick chorion that needs to be cut in order to pass a microinjection needle into the early blastomeres.

Injection techniques have also been developed for multiple *Euprymna* species using naturally laid embryos (Ahuja et al. 2023). The *Euprymna* chorion can be pierced directly with the micropipette once the jelly layers are manually removed. While *in vitro* fertilization is not currently established in *Euprymna*, the first cleavage takes place 8 h after fertilization, giving researchers a large window in which these embryos can be injected. Attempts to develop microinjection techniques have also been extended to several other cephalopod clades. While squid and cuttlefish embryos have similar extraembryonic structures (chorion, jelly layers, and tunic) (Lee et al. 2009; Montague et al. 2021), suggesting that microinjection should be broadly possible in this group, the extraembryonic structures in octopuses are quite different (Shigeno et al. 2015). Octopus embryos lack the jelly layers and tunic; instead, they have a very tough, leathery chorion, which may suggest that other approaches might be needed to deliver molecular reagents into cells in this group.

### CRISPRopods: creating knockouts and transgenics

Recent advances in CRISPR-based approaches have been applied in two different cephalopods (*D. pealeii* and *E. berryi*) to create knockout mutations. Two to three guides are designed per target using freely available programs such as CRISPRscan (Moreno-Mateos et al. 2015) or CHOPCHOP (Labun et al. 2019), and injected into early blastomeres, as described above. When injected into the first cell, >95% of alleles demonstrate a mutation in injected *D. pealeii* hatchlings, as shown by low-coverage amplicon sequencing (Crawford et al. 2020). G0 knockouts have been generated for species without a closed life cycle (e.g., *Doryteuthis pealeii*), which excludes the possibility of generating knockout lines. However, *Euprymna berryi* has been shown to have germline transmission, and several knockout lines have been established (Ahuja et al. 2023).

An important advance for the study of many biological questions will be the generation of transgenic or “knock-in” cephalopods, where a gene is introduced into the genome, such as a fluorescent reporter. There are several methods for inserting DNA into the genome of a research organism, including homology-directed repair (HDR) mediated via CRISPR-Cas (Sander et al. 2014), as well as random integration via restriction enzymes or transposases. While HDR via CRISPR offers precision in targeting the transgene to a particular locus, and can enable the exogenous gene to be expressed using the endogenous regulatory elements, the efficiency of integration is often low, and can be greatly influenced by the size of the insert. By contrast, integration via transposases, such as *tol2* (Kawakami et al. 1999), or restriction enzymes such as I-SceI meganuclease (Thermes et al. 2002), can be fairly efficient, but, as the integration of these constructs is random, the sequence for regulatory elements to drive expression must also be included. The production of transgenic cephalopods is an area of active research for several labs using different species and approaches, and will surely usher in exciting new insights into cephalopod biology.

### Target selection

A key first step in these molecular approaches is to identify target sequences for genetic manipulation. Annotation of protein-coding sequences in genome assemblies have provided a crucial first step, with resources currently being developed to identify regulatory elements. Several resources are now available to the cephalopod community to aid in this task, including long-read transcriptome sequencing for UTR detection, as well as ATAC-seq (Buenrostro et al. 2015) and DNA methylation (Fraga et al. 2002; de Mendoza et al. 2021; Macchi et al. 2022) methods for putative regulatory region annotation. Recent studies also employed HiC methods to detect genome topology (Schmidbaur et al. 2022). Ongoing community efforts include development of ChIP-seq methods and micro-C high-resolution HiC protocols. Additionally, several computational tools are available to predict regulatory elements based on databases of transcription factor (TF) binding profiles, such as Jaspar (Castro-Mondragon et al. 2022). These currently do not include mollusks or cephalopods, but comparative genomic data combined with published ATAC-seq and methylation data can be used to begin generating such resources. Further tools could be built to identify TF binding sites from gene expression lists for cephalopods (e.g., cistarget, Herrmann et al. 2012). Despite the availability of such tools and data, the bulk of the ongoing analyses are still performed on a gene-by-gene basis, involving manual curation and annotation. Combined RNA and ATACseq allows for identification of specific enhancers (González-Blas et al. 2022) in specific tissues or cells, and several methods, including scATAC-seq, are being developed for cephalopods. Additionally, using high-resolution topological information (micro-C) and single-cell data resolution, we may be able to disentangle gene regulation and identify specific regulatory regions—target gene associations.

## Advances in gene expression and single cell transcriptomics

Transcriptomics and spatial expression profile methods have been successfully applied to numerous emerging model organisms and hold great promise for the identification of genomic determinants putatively involved (and testable via transgenics) in anatomical novelties. While these tools have proven to be incredibly powerful in some species, their transfer to cephalopods has often been nontrivial. Below, we highlight the current state of cephalopod transcriptomics, including both the use of expression profiling, such as bulk RNA sequencing, single-cell RNA sequencing, and expression visualization, such as *in situ* hybridization and hybridization chain reaction (HCR). Together, these methods provide a more comprehensive view of cell types.

### Expression profiling

With a growing number of resources to explore cephalopod gene expression and regulatory elements (Crawford et al. 2020; Schmidbaur et al. 2022), uncovering the genetic bases underlying a cephalopod’s unique biological functions becomes increasingly feasible. Transcriptome sampling from diverse tissue types across cephalopod taxa ranges from neural systems (Albertin et al. 2015, 2022; Alon et al. 2015; Koenig et al. 2016; Liscovitch-Brauer et al. 2017) to microbe-associated, host-provided symbiotic organs (Castellanos-Martínez et al. 2014; Moriano-Gutierrez et al. 2019; Koch et al. 2020). Importantly, common RNA quality control metrics such as the RNA Integrity Numbers (RIN) cannot be used to assess cephalopod RNA quality. Mollusks (and most protostomes) possess a “hidden break,” where the 28S rRNA is cleaved into two similar-sized molecules, which leads to poor automatically assigned RIN values and a misinterpretation of the quality of the RNA (Natsidis et al. 2019). Sufficient cephalopod RNA integrity for next-generation sequencing should therefore be evaluated based on the presence of clear, narrow peak(s) and the absence of smearing.

Recent advances have enabled researchers to profile the transcriptomes of individual cells at a large scale. Single-cell RNA sequencing (e.g., 10× Genomics) has the potential to shed light on the molecular profiles of cephalopod cell types and to identify cell type-specific markers. The ability to distinguish cell types based on more than their morphology is an important step forward in the field and will facilitate the creation of specific transgenic reporter lines. Recently, cell type diversity has been described in the embryonic squid head (Duruz et al. 2022), the paralarval octopus brain (Styfhals et al. 2022), the octopus visual system (Songco-Casey et al. 2022), and the bobtail squid visual system (Gavriouchkina et al. 2022). The commonly used 10× Genomics Chromium Single Cell 3′ Kit, v3 chemistry, is 3′ biased such that the majority of reads are not counted when the genome annotation is suboptimal. Improving the annotation of the 3′ untranslated regions of current and future cephalopod genomes will lead to better mapping statistics and higher estimated numbers of sequenced cells (Lawson et al. 2020; Songco-Casey et al. 2022; Styfhals et al. 2022). Optimizing dissociation and culturing conditions for cephalopod cells will be important to improve cell viability and potentially library preparation. This is especially important, as many single-cell sequencing protocols have been developed for terrestrial and freshwater species, but do not account for the sensitivity of marine cells to changes in salinity. ScRNAseq studies in other marine organisms resuspend the cells in calcium/magnesium-free artificial seawater before proceeding to 10× Genomics (Cao et al. 2019; Sharma et al. 2019; Paganos et al. 2021). To circumvent potential problems relating to dissociation, viability, and sampling issues, single nuclei RNA sequencing provides a sound alternative.

### Expression visualization

The ability to localize RNA and proteins in cephalopods is an important tool in cephalopod genomics. RNA and localization in cephalopods have been executed for decades, beginning with the use of radiographical *in situ* hybridization (ISH) (Capano et al. 1987), and later with widespread use of colorimetric ISH utilizing anti-digoxygenin antibodies linked to enzymes that cleave colorimetric substrates (e.g., Shigeno et al. 2015; Tarazona et al. 2019). There has been a recent shift to the use of hybridization chain reaction fluorescent *in situ* hybridization (HCR-FISH) in cephalopod systems, including the localization of neuronal precursors (Deryckere et al. 2021; Elagoz et al. 2022) and localization of both host and symbiont factors in the bobtail squid light organ (Krasity et al. 2015; Nikolakakis et al. 2015), among other promising studies (Duruz et al. 2022; Gavriouchkina et al. 2022; Styfhals et al. 2022). As HCR-FISH utilizes self-amplifying fluorescent nucleotide hairpins, the technique allows for the cellular or subcellular localization of transcripts (Choi et al. 2020).

Immunocytochemistry (ICC) and immunohistochemistry (IHC) have also long been powerful tools for the cephalopod community. Due to the divergence between mammalian and cephalopod protein sequences, many cephalopod antibody studies employ custom antibodies, which, while effective, are expensive to produce and require effort to ensure specificity (Troll et al. 2009; Heath-Heckman et al. 2014; Li et al. 2019; Murata et al. 2021). Therefore, most studies use commercial antibodies on cephalopod tissue that cross-reacts to well-conserved proteins, peptides, or molecules (Di Cosmo et al. 2004; Springer et al. 2005; Goodson et al. 2006; Wollesen et al. 2010; Altura et al. 2011; Shigeno et al. 2015; Baldascino et al. 2017). While these protocols have been well established, there are still frontiers, such as the optimization of dual ICC/HCR protocols, either using established ICC protocols (Elagoz et al. 2022), or by new HCR–ICC techniques (Schwarzkopf et al. 2021). However, the lack of commercially available cephalopod-specific antibodies makes ICC out of reach for some research groups, and a centralized database of commercial antibodies that do work in cephalopod tissue could be an important resource for this burgeoning field.

### Steps to integrate gene expression and morphology

A multi-modal approach to integrate gene expression visualization and single-cell omics with other techniques promises to shed new light on cephalopod biology. By combining scRNAseq datasets with electron microscopy, we will be able to link the molecular information with their morphology and location, as was recently done for *Platynereis* (Vergara et al. 2021). Spatial transcriptomics also holds promise to detangle the cell type composition of cephalopods. Multi-ome sequencing approaches that can capture both gene expression and chromatin accessibility of individual cells will lead to a comprehensive database of cell-type-specific enhancers in cephalopods and characterize the epigenetic landscape.

Besides the interest in the developmental stages from embryos to adults in areas such as evolutionary developmental biology (evo-devo) or marine ecology, species such as *Octopus vulgaris* or *Sepioteuthis lessoniana* are highly attractive for human consumption and are a good candidate for aquaculture diversification. Transcriptome sequencing of early developmental stages cultured in different conditions are helping to the identification of biomarkers of growth, health, and welfare in aquaculture (García-Fernández et al. 2019; Prado-Álvarez et al. 2022; Varó et al. 2022). The identification and selection of biomarker genes will lead to the construction of specific q-PCR arrays as tools to analyze and guarantee welfare in aquaculture practices.

## Cephalopod metagenomics and microbial interactions

Yet another core aspect of cephalopod biology is how bacteria influence the development, evolution, and life history of the animal. Microbes have evolved along with their metazoan partners since their origin on Earth, and surveying their presence can inform us about host health (immunology and pathogen susceptibility), population dynamics, and the ecology of the association. Previous studies have demonstrated how bacteria influence specific developmental and behavioral features of cephalopods, and the cross-talk between partners (Nyholm et al. 2004, 2021; Visick et al. 2021). Over the past few decades, much of the experimental work on cephalopod microbiomes has been focused on monotypic associations, including the light organ symbiosis in sepiolid and loliginid squids, and complex microbiomes, such as the accessory nidamental glands (ANGs) in the Decapodiformes (Kaufman et al. 1998; Barbieri et al. 2001; Nyholm et al. 2021). Both of these microbial systems have been important models for understanding how bacteria influence development of the specific organs they are housed in, as well as their ability to produce antimicrobial/antifungal compounds found in newly laid egg masses (Barbieri et al. 1997; Kerwin et al. 2019). Microbial communities have also been characterized in the gut of cuttlefish (*Sepia*) and wild octopus (*O. vulgaris*), but very little is known about other communities of bacteria that reside on or inside cephalopods (Roura et al. 2017; Lutz et al. 2019). Lastly, host specificity, and the variation among environmentally transmitted bacteria throughout populations of cephalopods has illuminated our comprehension of microbial diversity, coevolution between the partners, and microbe-microbe interactions prior to and during the association (Jones et al. 2006; Guerrero-Ferreira et al. 2013; Coryell et al. 2018).

### Modes of microbial associations in cephalopods

The symbiotic relationship of bioluminescent *Vibrio fischeri* and cephalopods have been studied for many decades (Nyholm et al. 2021). Different molecular tools are used to experimentally analyze the interaction of the light organ in *E. scolopes* and *V. fischeri*. Further, *Vibrio*-specific 16S rRNA primers are available to identify different *Vibrio* species in different cephalopods (Nishiguchi et al. 2003; Zamborsky et al. 2011; Howard et al. 2015). The interrogation of both the light organ and ANG in *E. scolopes* has the potential to reveal common and unique mechanisms by which hosts regulate different microbial niches (Nishiguchi et al. 2004; Nyholm and McFall-Ngai 2021). We can use 16S rRNA to identify bacteria in different species of cephalopods and in different organs. The ANG microbiome (Barbieri et al. 2001; Pichon et al. 2005; Kerwin et al. 2017, 2018; Yang et al. 2021), along with the microbiota of gills, skin, and the digestive system (Roura et al. 2017; Lutz et al. 2019; Farto et al. 2019; Hanlon, Forsythe 1990), have been characterized in some squid, cuttlefish, and octopus (Roura et al. 2017; Lutz et al. 2019; Kang et al. 2022). A core microbiome based on taxonomy has not been identified, but there is potential to identify fundamental functional microbiomes in different organs.

Several methods have been employed to characterize microbiome interactions in the model cephalopod *E. scolopes*, such as 16S rRNA gene diversity metagenomics, transcriptomics/RNA-seq, genomics, proteomics, and metabolomics (Collins et al. 2012a; Altura et al. 2013; Bongrand et al. 2016, 2020; Kerwin et al. 2018; Belcaid et al. 2019; Moriano-Gutierrez et al. 2019; Koch et al. 2020) and techniques, such as HCR-FISH, FISH, immunofluorescence, and fluorescently tagged bacteria, were used to visualize host-bacteria interactions in *E. scolopes* (Troll et al. 2010; Collins et al. 2012a; Nikolakakis et al. 2015). Macrophage cells (hemocytes) can be easily obtained and observed for their interactions with symbiotic and non-symbiotic bacteria with microscopy (Nyholm et al. 2009; Rader et al. 2019) and at the molecular level with proteomics and transcriptomics (Collins et al. 2012b; Schleicher et al. 2014).

Recent technological advancements permit finer-resolution studies of population genetic structures that will be relevant to symbiotic association analyses. Double digest restriction site-associated DNA sequencing (ddRADseq) is a low-cost, high-throughput technique in genotyping thousands of single nucleotide polymorphisms (SNPs) in any species, including non-model organisms like cephalopods (Andrews et al. 2016). This reduced-representation sequencing approach could be powerful for deducing population genetic structures of the host cephalopods and obtain first indication of microbial species present. Since abiotic factors like temperature, current, and salinity have been found to influence the movement and concentrations of *Vibrio* spp. in sepiolids (Nishiguchi 2000; Jones et al. 2006; Soto et al. 2009; Nourabadi et al. 2021), understanding how the population genetic structures of the host relate to that of the symbionts could be crucial in discerning their population ecology and dynamics with important evolutionary outcomes (Pankey et al. 2014). SNP datasets obtained from ddRADseq and other metagenomic (e.g., bulk sequencing) approaches on host and symbiont would allow further research into the associations between adaptive genetic markers (either unique or shared between hosts and symbionts) and environmental factors, thereby identifying potential selection pressures in particular populations (Lindgren et al. 2012).

### Emerging directions in cephalopod symbiosis research

Experimentally tractable model systems have been critical for understanding the influence of microbiomes on eukaryotic biology (Douglas 2019; McFall-Ngai et al. 2021). Having the ability to generate axenic/germ-free hosts or hosts with a known microbiota (gnotobiotic) have led to groundbreaking discoveries in vertebrate host-microbe systems, e.g., showing linkages between the microbiome and obesity, type 2 diabetes, autism, and behavior. Developing gnotobiotic and germ-free aquatic husbandry systems would allow for the further expansion of the use of cephalopods in microbiome studies. One challenge in microbiome research is studying direct interactions between host and microbial cells. Cell culture of cephalopod tissue types like the mucosal epithelia of organs that directly interact with symbionts and innate immune cells (hemocytes) would help reveal the molecular cross-talk between the partners. Single-cell multi-omic methods could then also be applied to these systems to identify the role of cell-cell interactions in cephalopod symbioses.

Genomic and metagenomic analysis of microbial communities shed light on microbiome function, e.g., by revealing important metabolic pathways, signaling pathways, and effectors that influence host biology. In order to expand the study of cephalopod microbiomes, it will be critical to increase the number of symbiont reference genomes that are available for metagenomic and metatranscriptomic datasets. Having better reference genomes will also allow for linking biosynthetic gene clusters (BGCs) to the production of specific compounds that can be identified from metabolomics studies.

Studies investigating how cephalopod microbiomes change over time, and whether these changes can give us a window to the temporal dynamics of the host would lead to a greater understanding of how host-microbe associations are initiated, persist, and evolve over time. This includes experimental evolution studies, where microbes are evolved in a novel host species to determine which genetic cues are responsible for recognition and specificity (Soto et al. 2012, 2014a, b). Interestingly, this type of work has already been shown to be important in human microbiome research (Bosch et al. 2019; Burnetti et al. 2022; Garud 2022); having a temporal framework of these microbial populations would further our knowledge of the importance of bacteria in cephalopod health (Lynch et al. 2019). Given that much of the applied work in cephalopod husbandry and aquaculture is focused on preventing disease, knowledge of the balance between beneficial and pathogenic microbial species will be important for the future success of expanding these important fisheries.

## Conclusions and future directions: CephBase and best practices for sharing resources

We are at an exciting and critical time in the evolution of the cephalopod field, with many new resources and techniques becoming available that will allow us to address fundamental questions of cephalopod biology and evolution. As the pace and complexity of generated datasets for cephalopods is intensifying, an emerging community goal will be focused on data integration for cephalopod-omics, including but not limited to the creation of shared databases for genomes, annotations, expression data, as well as cultures. Current efforts are emerging from individual labs, e.g., the Cephalopod Program at the Marine Biological Laboratory, which is a resource for genetically tractable lines and developing genetic tools for transgenics (https://www.mbl.edu/research/resources-research-facilities/marine-resources-center/cephalopods/cephalopod-breeding-center), *Euprymna* genomics portal (https://metazoa.csb.univie.ac.at/), and CephRes (www.cephalopodresearch.org), among others. While efforts are being made to make a repository of tractable cephalopod species, lab contacts, their techniques, as well as available resources (see below), a consolidated effort would require funding sources and is otherwise restricted by “good-will,” research grants to individual labs, or philanthropy.

Therefore, as the cephalopod community continues to grow, we propose several steps to ensure that the community can share resources effectively. The first resource that is important to share is genomic and transcriptomic data. Drawing on successful approaches from the *C. elegans* (wormbase) and the *D. melanogaster* (flybase) communities, we would like to establish a centralized database for cephalopod bioinformatic data. A simple user interface will allow researchers to compile the information they need more easily, including expressions and verified functional annotations. This repository could also serve as a means to share techniques and protocols. To complement this, any plasmids used for these techniques should be deposited to Addgene, or other repositories. Importantly, the source of the genomic information (such as the geographic location of sampling), is crucial for traceability (Vecchione et al. 2022). The third resource that is important to share is animals, both wild types and, eventually, transgenics. Each cephalopod species has many unique characteristics that makes them ideal for answering different questions, and thus no one species has emerged as the main species within the cephalopod research community. This comes with drawbacks, e.g., a technique that works in squid may not work in octopus. To take advantage of the opportunities that will emerge from a field with a strong comparative element, our longer-term vision includes dedicated cephalopod rearing facilities and centers of excellence for specific techniques and species around the world. This will encourage collaboration and allow scientists to travel to learn/perform specialized experiments on specific species.

The first attempts have been made to molecularly characterize cephalopod cell types at different scales (organs, systems, and whole organisms). A large collaborative effort is required to construct a “Tabula Cephalopodae” to obtain a complete taxonomy of cephalopod cell type diversity. By bringing together the expertise of different research groups, we hope to streamline future studies and attempt to combine these datasets in a single-cell cephalopod atlas. By including multiple key cephalopod species, we can examine clade-specific novelties such as different brain lobes and the light organ and shed light on cell-type evolution across cephalopods.

Our community has also developed further momentum to go beyond a single-organism focus. As outlined above, disruption of the microbiome’s integrity is a mechanism by which stress affects animal health and welfare (Uren Webster et al. 2020). Climate change increases ocean warming and acidification, which constitutes a stressful condition (Apprill 2020; Bénard et al. 2020). Additionally, the intensification of aquaculture is associated with challenges regarding its sustainability, including its impact on animal health and welfare, the environment, food safety, and economic viability. Intensive aquaculture can expose cephalopods to crowding, handling, and social stressors, which can induce negative effects on health and fitness, such as impaired growth, depressed immunity, and increased susceptibility to disease. Besides developing juveniles and adults, early life stages of cephalopods are particularly sensitive to environmental stressors, and there is increasing recognition that microbial communities may contribute to harmful effects (Roura et al. 2017). To this end, the continued analysis of gut, skin, or fecal microbiomes can be of great interest to monitor the stress response and, therefore, to assess the welfare and health status of cephalopods. Linking-omic datasets for cephalopods and their metagenomes, including data from bacteria, archaea, fungi, and bacteriophages (Koskella et al. 2013; Guerin et al. 2020), can thus also aid in disease identification in aquaculture and the natural environment.
